# Association of RXR-Gamma Gene Variants with Familial Combined Hyperlipidemia: Genotype and Haplotype Analysis

**DOI:** 10.1155/2013/517943

**Published:** 2013-10-13

**Authors:** Federica Sentinelli, Ilenia Minicocci, Anna Montali, Luisa Nanni, Stefano Romeo, Michela Incani, M. Gisella Cavallo, Andrea Lenzi, Marcello Arca, Marco G. Baroni

**Affiliations:** ^1^Endocrinology and Diabetes, Department of Experimental Medicine, Sapienza University of Rome, 00161 Rome, Italy; ^2^Endocrinology and Diabetes, Department of Medical Sciences, University of Cagliari, 09042 Cagliari, Italy; ^3^Department of Clinical and Medical Therapy, Unit of Atherosclerosis, Sapienza University of Rome, 00161 Rome, Italy; ^4^Sahlgrenska Center for Cardiovascular and Metabolic Research, Department of Molecular and Clinical Medicine, University of Gothenburg, SE-413 45 Gothenburg, Sweden

## Abstract

*Background*. Familial combined hyperlipidemia (FCHL), the most common genetic form of hyperlipdemia, is characterized by a strong familial clustering and by premature coronary heart disease. The FCHL locus has been mapped to human chromosome 1q21-q23. This region includes the retinoid X receptor gamma (RXRG), a nuclear factor member of the RXR superfamily, which plays important roles in lipid homeostasis. *Objective*. To investigate the possible role of the RXRG gene in the genetic susceptibility to FCHL. *Methods*. Variations in RXRG gene were searched by direct sequencing, and the identified SNPs were genotyped by PCR-RFLP in 192 FCHL individuals from 74 families and in 119 controls. *Results*. We identified 5 polymorphisms in the RXRG gene (rs1128977, rs2651860, rs2134095, rs283696, and rs10918169). Genotyping showed that the A-allele of rs283696 SNP was significantly associated with FCHL (corrected *P*, *P*_*c*_ < 0.01). Also the alleles of the rs10918169 and of the rs2651860 SNP were more frequent in FCHL subjects compared to those in controls, although not significantly after correction. When the clinical characteristics of the FCHL subjects were stratified by allele carrier status for each SNP, the rs2651860 SNP was significantly associated with increased levels of LDL-cholesterol and of Apo-B in T-allele carriers (*P* < 0.04). Finally, haplotypes analysis with all 5 SNPs confirmed the significant association of RXRG gene with FCHL. Specifically, the haplotype containing all 3 “at-risk” alleles, significantly associated with FCHL (A-allele of rs283696, G-allele of rs10918169, and T-allele of rs2651860), showed an OR (Odds Ratio) of 2.02, *P*_*c*_ < 0.048. Conversely, the haplotype without all these 3 alleles was associated with a reduced risk for FCHL (OR = 0.39, *P*_*c*_ < 0.023). The “at-risk” haplotype CTTAG was also associated with higher LDL-C (*P* < 0.015). In conclusion, variation in the RXRG gene may contribute to the genetic dyslipidemia in FCHL subjects.

## 1. Background

Familial combined hyperlipidemia (FCHL) is the most common atherogenic disorder of lipid metabolism [1, 2], typically characterized by multiple hyperlipemic phenotypes within the same individual as well as in the same family, where elevated very-low-density lipoproteins (VLDL) and/or low-density lipoproteins (LDL) or apolipoprotein B (apoB) can be detected [3–5].

FCHL shows strong genetic susceptibility and an autosomal dominant mode of inheritance with low penetrance [1, 6]. However, most of the underlying genes remain to be elucidated.

A previous linkage analysis with markers from ten chromosomal regions that contain lipid-metabolism candidate genes revealed that one marker (D1S104) on chromosome 1 yielded a LOD score of 3.5. A multipoint analysis combining information from D1S104 and the neighboring marker D1S1677 resulted in a LOD score of 5.93 [7]. These data strongly supported the notion of an FCHL gene on chromosome 1q.

Previous studies have shown that nuclear receptors, especially retinoid X receptors (RXR) and its heterodimerization partners, play important roles in maintenance of lipid homeostasis on their activation by a variety of ligands derived from dietary cholesterol and fatty acids [8, 9].

Furthermore, Knoblauch et al., observed that the retinoid X receptor (RXR) gene locus is linked to total cholesterol (*P* < 0.001), LDL (*P* < 0.004), and triglyceride (*P* < 0.0004) concentrations in an identical-by-descent (IBD) linkage analysis of normal dizygotic twin subjects and their parents [10]. 

The human RXRG gene is located in chromosome 1q21-q23, the so-called “FCHL locus” [7], so this gene is an interesting candidate for involvement in FCHL.

In this study we have searched for variations within the DNA sequence of the RXRG gene in subjects with FCHL phenotype and controls, and subsequently analysed their association with FCHL.

## 2. Methods

### 2.1. Study Population

Seventy-four Italian families with FCHL were enrolled in the study. They were identified through probands (*n* = 74) diagnosed according to previously reported criteria [5, 11]. Briefly, FCHL probands were required to be >20 years of age, to have total cholesterol (TC) and/or triglycerides (TG) levels greater than or equal to those of age- and sex-specific 90th percentiles in the Italian population, to have isolated elevation of plasma apoB concentrations (>130 mg/dL corresponding to the 90th Italian population percentile), and to have at least one first degree relative with hyperlipidemia (TC and/or TG > 90th percentile). A total cohort of 192 subjects with FCHL was enrolled. Among probands' relatives, 118 were defined as affected by FCHL according to the above criteria. Individuals with obesity (body mass index (BMI) > 30 Kg/m^2^) or poorly controlled diabetes mellitus (blood glucose > 120 mg/dL and/or glycosylated hemoglobin > 6.0%) were excluded. Other acquired causes of hyperlipidemia (including thyroid and liver disease, renal failure, and proteinuria) were excluded by standard laboratory tests.

Control subjects (*n* = 119) were randomly selected from normolipemic subjects participating in a community-based screening for coronary risk factors. All subjects were unrelated, and exclusion criteria were diabetes mellitus (history of hypoglycemic treatment and/or fasting blood glucose >126 mg/dL) or current treatment with lipid-affecting drugs and no family history of hyperlipidemia.

The Sapienza University of Rome ethical committee approved the study protocol, and all subjects provided their written informed consent to participate into the study.

The clinical and biochemical characteristics of probands, all FCHL patients, and controls are reported in Table 1.

### 2.2. Laboratory Analysis

Glucose, insulin, cholesterol, HDL-cholesterol, and triglycerides (TGs) were measured after an overnight fast.

Plasma and lipoprotein fractions were assayed for total cholesterol and triglycerides using enzymatic reagents; high-density lipoprotein cholesterol (HDL-C) was determined after precipitation of apoB-containing lipoproteins as reported [13]. Low-density lipoprotein cholesterol (LDL-C) was estimated by the Friedewald's formula, except when plasma triglyceride concentration exceeded 400 mg/dL (4.52 mmol/L). Total plasma apolipoprotein B (apoB) was measured by immunoturbidimetric method (Kone Instruments, Espoo, Finland). Plasma concentrations of apoB were not available for normolipemic controls.

Fasting plasma glucose was measured with a glucose oxidase method.

Fasting plasma insulin concentrations were measured using a radioimmunoassay kit (Biodata Insulin Kit, Milan, Italy) with a 7.5% interassay variation coefficient. 

### 2.3. Molecular Genetic Screening

Blood specimens were collected in EDTA-containing tubes, and genomic DNA was prepared from leukocytes using the conventional salting out extraction method.

The human RXRG gene (GeneBank accession n. NG_029517) is encoded in a 43.8-kb segment on chromosome 1q22-23, and the 1392 nucleotide transcript is contained within 10 exons. 

Ten primer pairs were used to amplify the 10 exons, the 5′ regulatory region, and the 3′-untranslated region (3′-UTR). Primers were designed to include intron-exon boundaries, in order to evaluate possible mutations in splicing sites. A highly conserved intronic region (identified from UCSC (University of California, Santa Cruz) genome browser) next to exon 5 was also sequenced. 

The RXR-gamma gene locus was sequenced in all 74 FCHL probands and in 50 controls. 

**Figure 1:**
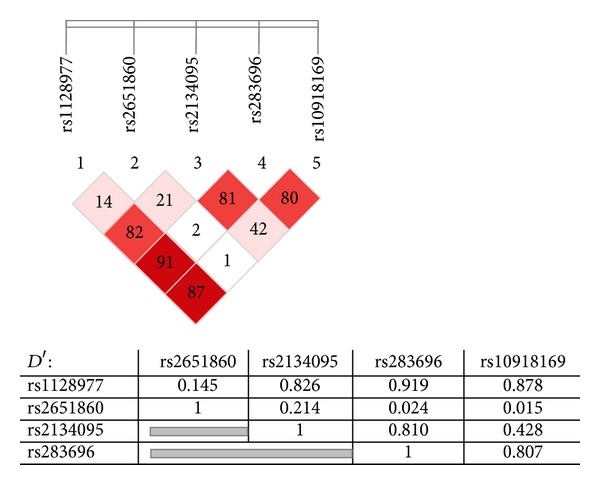
Linkage disequilibrium test (*D*′) of the five SNPs (single nucleotide polymorphisms) in retinoid X receptor-gamma (RXR-gamma) gene. Dark red diamonds represent high association (*D*′ ≥ 0.87), moderate red represent *D*′ between 0.83 and 0.81, and light red represent *D*′ of 0.73. Table shows *D*′ values.

We selected for genotyping, 5 of the SNPs (single nucleotide polymorphisms) identified by sequencing, among those with a minor allele frequency (MAF) >0.20. Nonrare variants were detected below this MAF. The identified SNPs were studied in the whole population by Polymerase Chain Reaction-Restriction Fragment Length Polymorphism (PCR-RFLP). In particular restriction enzymes Hpy 99-I for rs1128977, NlaIII for rs2651860, HinfI for rs2134095, NlaIII for rs283696, and HinfI for rs10918169 were used for genotyping. The relative position of these five SNPs and their linkage disequilibrium are shown in Figure 1.

### 2.4. Statistical Analysis

Data are expressed as means ± standard deviations. Categorical variables were analysed by *χ* ^2^ or Fisher's exact tests. Continuous variables were analysed by Student's *t*-test and Mann-Whitney test. Hardy-Weinberg equilibrium was assessed by *χ* ^2^ analysis. Genotype and allele frequencies were calculated by the software SHEsis (http://analysis.bio-x.cn/myAnalysis.php/) and compared between cases and controls by a two-sided Fisher exact test. A *P* < 0.05 was considered significant.

Haplotypes in cases and controls, as well as Lewontin's *D*′ as a measure of linkage disequilibrium (LD) between paired SNPs, were also estimated using the SHEsis program which determines the most probable haplotypes based on allele frequencies. Haplotypes with a frequency of < 0.03 were excluded from the analysis. The SHEsis program was used to calculate the odds ratios (OR) and 95% confidence intervals (95%CI) for FCHL associated with each haplotype. Haplotypes predicted by SHEsis were associated with lipid phenotypes by evaluating changes of lipids levels for at least one copy of tested haplotype against reference haplotypes. These comparisons were carried out using the Student's *t* and Mann-Whitney tests. 

For the genetic analyses we took into consideration *P* values after correction for multiple comparisons (*P*_*c*_), calculated on the number of SNPs in our study. A *P*_*c*_ value <0.05 was considered statistically significant. However, adjustment for multiple comparisons (Bonferroni's correction) has been shown to, in some cases, be too conservative, be valid for testing the universal null hypothesis, and to increase the chance for type II errors [14]. 

Except for haplotype data, all statistical analyses were performed with SPSS (Statistical Product and Service Solutions) 17.0 statistical package.

## 3. Results

### 3.1. Clinical Characteristics of Study Subjects

Table 1 compares the clinical characteristics of the study groups. Analyses in FCHL patients, either including all affected subjects or only the probands, showed significantly lower HDL-C (*P* < 0.001), higher TC (*P* < 0.001), LDL-C (*P* < 0.001), and TG (*P* < 0.001) in FCHL subjects compared to controls, as expected. 

HOMA-index and insulin levels were higher in controls although not significantly different among the study groups.

### 3.2. Association Analyses of RXRG Gene Variants

We searched for variants by direct PCR sequencing the ten exons of the RXRG gene, together with its 5′ regulatory region and 3′-UTR in all 74 affected FCHL subjects and in 50 control subjects. We identified five sequence variations with a MAF > 0.20: exon 3 C > T (rs1128977), the intronic region 4 T > G (rs2651860), exon 8 T > C (rs2134095), the intronic region 9 G > A (rs283696), and 3′-UTR G > C (rs10918169). The two SNPs found in exons 3 and 8 did not alter the amino acid sequence (Supplementary Table 1, see Supplementry Material available online at http://dx.doi.org/10.1155/2013/517943). Two other SNPs with a MAF = 0.10 were identified (rs3753897 and rs185905), and for this reason they were not studied in the whole population.

The genotypes frequencies did not show a significant deviation from the Hardy-Weinberg equilibrium. In addition, no effect of age, sex, or BMI on their distribution was observed in FCHL subjects and controls (data not shown).

We thus evaluated the genotype and allele frequencies of the 5 identified polymorphisms within the RXRG gene in FCHL probands (*n* = 74), in all affected FCHL subjects (*n* = 192, including the 74 probands and 118 relatives) and in 119 controls (Table 2). 

The A-allele of the RXRG rs283696 variant was found to be more frequent in FCHL probands compared to controls (0.32 and 0.22 resp., *P*_*c*_ = *NS*, corrected *P* nonsignificant) and also significantly more frequent in all FCHL affected subjects compared to controls (0.33 and 0.22 resp., *P*_*c*_ < 0.01). Also, the G-allele of the rs10918169 SNPs was more frequent in all FCHL affected compared to control subjects (0.32 and 0.24 resp., *P*_*c*_ = NS). Finally, genotype distributions of the rs2651860 SNP differed in both FCHL probands and all FCHL affected subjects compared to controls (*P*_*c*_ = NS). 

### 3.3. Phenotypic Characterisation of Risk-Allele Carriers

To establish the impact of the identified RXRG variants on metabolic parameters, we evaluated the clinical characteristics of the FCHL probands and of all FCHL affected subjects stratified by allele carrier status for each SNP. Only the rs2651860 SNP showed a significant association with increased levels of LDL-C (169 ± 44 versus 141 ± 57 mg/dL, *P* < 0.026 after adjustment for BMI, age, and sex) and of Apo-B (160 ± 31 versus 141 ± 34 mg/dL, *P* < 0.039 after adjustment for BMI, age, and sex) in T-allele carriers. All other SNPs did not show any significant association (data not shown). 

The same analyses were performed in all FCHL affected group. Again, individuals carrying the T-allele of the rs2651860 SNP showed significantly higher levels of LDL-C (161 ± 47 versus 149 ± 47 mg/dL, *P* < 0.049 after adjustment for BMI, age, and sex). 

### 3.4. Haplotype Analysis of the RXRG Gene

Based on genotypes data, we found that the rs1128977, rs2134095, rs283696, and rs10918169 SNPs were highly correlated (Lewontin's *D*′ between 0.807–0.919), with the rs1128977 and rs283696 being particularly close (*D*′ > 0.91) (Figure 1). 

The haplotypes reconstruction by SHEsis program generated 25 haplotypes; 16 were rarer (frequency < 0.03) and were, then, excluded from the analysis. The frequencies of the remaining 9 haplotypes were compared in all FCHL patients and controls (Table 3). Two haplotypes (CTTAG and TGTGC) showed significant difference in frequencies between all affected and controls. In particular, haplotype CTTAG was significantly more frequent in all FCHL patients than controls with an OR of 2.02 (CI = 1.13–3.59, *P*_*c*_ < 0.048), and haplotype TGTGC was significantly underrepresented among patients compared to controls with an OR of 0.39 (CI = 0.19-0-76, *P*_*c*_ < 0.023). Hence, the TGTGC haplotype resulted to be significantly associated with a 61% lower risk of FCHL.

We also evaluated haplotype frequencies only in the FCHL probands for the five SNPs. The two significant haplotypes in the previous analysis (CTTAG and TGTGC) showed the same trend as before, although not reaching statistical significance, possibly because of the smaller number of cases. These haplotype frequencies were equivalent to those observed in the whole FCHL cohort (data not shown). 

To evaluate the effects of haplotypes on lipids, we compared, in the whole group of FCHL patients and controls, lipid parameters in carriers of the “risk” haplotype CTTAG against those carrying all other haplotypes. The results of this analysis showed that individuals carrying the risk haplotypes had significantly higher LDL-C (154 ± 53 mg/dL versus 139 ± 41 mg/dL, *P* = 0.015) than subjects with all other haplotypes (data not shown).

These differences were not related to change in body fat or age, as mean BMI and age values were almost similar between the two groups. 

## 4. Discussion

In this study we have investigated the possible role of the RXRG gene in the genetic susceptibility to FCHL. We analysed the potential association of 5 genetic variants in the RXRG gene that were identified by direct sequencing in Caucasian population of FCHL subjects and controls.

We observed a significant association between one of the SNPs in the RXRG gene and FCHL, specifically SNP rs283696, and an increased frequency of s2651860 and rs10918169 SNP in FCHL subjects. Furthermore, haplotypes constructed with all 5 SNPs confirmed the significant association of RXRG with FCHL. In particular the haplotype containing all 3 “at-risk” alleles that were more frequent in FCHL (T-allele of rs2651860, A-allele of rs283696, and G-allele of rs10918169) showed an OR of 2.02, *P* < 0.048. Conversely, the haplotype without all these 3 alleles was associated with a reduced risk for FCHL (OR = 0.39, *P* < 0.023). The “at-risk” haplotype CTTAG was also associated with higher LDL-C (*P* < 0.015). The observation that the CTTAG haplotype is more strongly associated with FCHL than the single alleles of each SNP suggests an additive effect on the association given by all “at-risk” alleles. Although some initially significant results changed to nonsignificant after correction for multiple comparisons, several results remained statistically meaningful, including the association between FCHL with SNP rs283696 and with the “at-risk” CTTAG and “protective” TGTGC haplotypes, showing that these associations are consistent. 

We also looked at the possible impact of the identified RXRG variants on metabolic parameters, evaluating the clinical characteristics of the FCHL probands and of all FCHL affected subjects stratified by allele carrier status for each SNP. Only the rs2651860 SNP showed a significant association with elevated LDL-C and ApoB in FCHL subjects, suggesting a possible role in the determination of this trait. However, this association warrants further replication since the corrected *P* value was nonsignificant.

Any of the sequence variants identified and tested in our study alter the coding sequence of RXRG gene. However, the three SNPs associated with FCHL are intronic or in the 3′ UTR, therefore possibly affecting regulatory regions. We can hypothesize that these SNPs may alter RXRG gene expression or might be in strong linkage disequilibrium with undetected noncoding variants, which in turn may alter gene expression.

A previous study in Japanese subjects reported significant association between a SNP (Gly14Ser in exon 1) in RXRG gene and FCHL [15]. However, this G502A variation reported in Nohara et al. was found in none of our individuals (including control subjects, data not shown), possibly because it is restricted to Asian populations. Nevertheless, both our results and those presented in Nohara's study identify in the RXRG gene, or in a locus in linkage with it, a possible genetic determinant of FCHL.

A few limitations have to be considered in interpreting these results. It may be observed that the significant association between RXRG haplotypes and FCHL risk might be biased by the inclusion of related individuals. However, since the frequency of the “at-risk” haplotype remained higher and equivalent in FCHL probands than in controls, this suggests a noncausal haplotype distribution. We also acknowledge that the present findings have not been replicated in an independent group of FCHL subjects, but we would like to point out the strict diagnostic criteria for FCHL that limit the recruitment of other cohorts. Nonetheless, we interpret our observations as a support to previous findings indicating the presence of a gene for FCHL on chromosome 1q [15, 16], with strong signals supporting the RXRG gene.

The RXRG is one of the members of the steroid/thyroid hormone superfamily of nuclear receptors that as transcription factors plays unique modulatory and integrative roles across multiple metabolic systems. 

RXRG is expressed mostly in brain, muscle, skin, intestine, lung, and adipose tissue [17]. In animal models, RXRG knock-down mice survive and develop apparently normally [18]. Haugen et al. observed that mice lacking RXRG had increased metabolism, decreased plasma TGs, increased skeletal muscle LPL activity, and resistance to weight/fat mass gain when challenged with a high fat (HF) diet [19]. 

RXRG heterodimerizes with the farnesol X receptor (FXR) [or bile acid receptor (BAR)], the liver X receptors (LXRs), and the peroxisome proliferator-activated receptors (PPARs). Heterodimers between RXR and PPAR*γ*, PPAR*α*, LXR*α*, and FXR/BAR, respectively, influence glucose, triglyceride, cholesterol, and bile acid homeostasis. Dysregulation of these homeostatic control pathways can result in metabolic disorders such as obesity, type 2 diabetes, and hyperlipidemia, which are often complicated by the development of atherosclerosis [20].

In particular, once LXRs form a heterodimer with RXR, they stimulate the transcription of an array of genes involved in the absorption, efflux, transport, and excretion of cholesterol and other lipids [21]. Claudel and coworkers proved the importance of the LXR/RXR heterodimer in the induction of cholesterol efflux from macrophages. The defective activation of cholesterol efflux by the LXR/RXR heterodimer might therefore contribute to the effects of RXRG gene on dyslipidemia.

## 5. Conclusion

The present study suggests that variation in the RXRG gene may contribute to the genetic dyslipidemia in FCHL subjects, suggesting a possible genetic role in determining this trait.

## Supplementary Material

Supplementary Table 1 shows the five genotyped SNPs of the RXR-gamma gene and their minor allele frequencies (MAF) in our controls.

## Figures and Tables

**Table 1:** Clinical characteristics of FCHL patients and controls.

	Controls (*n* = 119)	FCHL	
		Probands (*n* = 74)	All affected (*n* = 192)
Age (yrs)	51.96 ± 12.10	47.77 ± 12.47*	49.81 ± 14.83
Sex (M/F)	53/66	50/24**	111/81*
BMI (Kg/m^2^)	24.83 ± 3.69	26.47 ± 3.64**	25.96 ± 3.71*
Current smokers, *n* (%)	—	22 (29.7%)	54 (28.1%)
Hypertension, *n* (%)	27 (22.7%)	14 (18.9%)	37 (19.3%)
Blood glucose (mg/dL)	92.97 ± 18.20	89.13 ± 14.64	87.98 ± 13.15**
Insulin (mUI/L)	16.76 ± 19.18	11.70 ± 8.51	11.41 ± 6.86
HOMA index	3.53 ± 4.14	2.59 ± 2.17	2.49 ± 1.77
Diabetes, *n* (%)	4 (3.4%)	2 (2.7%)	6 (3.1%)
CAD, *n* (%)	—	11 (14.9%)	29 (15.1%)
Plasma lipids (mg/dL)			
TC	199.95 ± 37.50	252.95 ± 56.07***	251.61 ± 46.25***
HDL-C	56.93 ± 13.60	45.95 ± 13.93***	48.21 ± 14.42***
TG	104.81 ± 56.72	270.64 ± 131.77***	246.91 ± 128.59***
LDL-C	122.05 ± 32.3	155.58 ± 52.5***	154.93 ± 46.80***
ApoB	—	151.09 ± 33.56	155.61 ± 30.78

Data are reported as means ± SD.

BMI: body mass index; HOMA: homeostasis model assessment calculated according to Matthews et al. [12]; CAD: coronary artery disease; TC: total cholesterol; HDL-C: high density lipoprotein cholesterol; TG: total triglycerides; LDL-C: low density lipoprotein cholesterol; ApoB: apolipoprotein B; SD: standard deviation; FCHL: familial combined hyperlipidemia; M: male; F: female.

Probands were index cases of each 74 FCHL kindred.

*P < 0.05, **P < 0.005, and ***P < 0.001 for comparison between controls and probands or all FCHL patients.

**Table 2:** Genotype and allele frequencies of individual SNPs within RXR-gamma gene in FCHL probands and controls.

SNPs	Controls (*n* = 119)	Probands (*n* = 74)	*χ* ^2^	Fisher's *P*	OR (95% CI)	All affected (*n* = 192)	Fisher's *P*
rs1128977	(*n* = 97)	(*n* = 70)				(*n* = 177)	
C25464C	40 (0.41)	25 (0.36)	1.28	0.53		68 (0.38)	0.17
C25464T	40 (0.41)	35 (0.50)				90 (0.51)	
T25464T	17 (0.18)	10 (0.14)				19 (0.11)	
Allele C	120 (0.62)	85 (0.61)	0.04	0.83	0.95 (0.61–1.49)	226 (0.64)	0.64
Allele T	74 (0.38)	55 (0.39)				128 (0.36)	
rs2651860	(*n* = 106)	(*n* = 73)				(*n* = 188)	
T33538T	37 (0.35)	37 (0.51)	5.47	0.24*		85 (0.45)	0.23*
T33538G	40 (0.38)	17 (0.23)				46 (0.25)	
G33538G	29 (0.27)	19 (0.26)				57 (0.30)	
Allele T	114 (0.54)	91 (0.62)	2.59	0.11	0.70 (0.46–1.08)	216 (0.57)	0.39
Allele G	98 (0.46)	55 (0.38)				160 (0.43)	
rs2134095	(*n* = 106)	(*n* = 74)				(*n* = 189)	
T37041T	59 (0.56)	46 (0.62)	0.83	0.66		114 (0.60)	0.58
T37041C	41 (0.39)	25 (0.34)				62 (0.33)	
C37041C	6 (0.06)	3 (0.04)				13 (0.07)	
Allele T	159 (0.75)	117 (0.79)	0.80	0.37	0.79 (0.48–1.31)	290 (0.77)	0.64
Allele C	53 (0.25)	31 (0.21)				88 (0.23)	
rs283696	(*n* = 116)	(*n* = 73)				(*n* = 183)	
G38550G	72 (0.62)	35 (0.48)	5.08	0.08		88 (0.48)	0.05*
G38550A	38 (0.33)	29 (0.40)				68 (0.37)	
A38550A	6 (0.05)	9 (0.12)				27 (0.15)	
Allele G	182 (0.78)	99 (0.68)	5.32	0.1*	1.73 (1.08–2.76)	244 (0.67)	**0.01**
Allele A	50 (0.22)	47 (0.32)				122 (0.33)	
rs10918169	(*n* = 118)	(*n* = 72)				(*n* = 168)	
G44118G	9 (0.08)	9 (0.12)	2.77	0.25		21 (0.12)	0.13
G44118C	38 (0.32)	28 (0.39)				65 (0.39)	
C44118C	71 (0.60)	35 (0.49)				82 (0.49)	
Allele G	56 (0.24)	46 (0.32)	3.07	0.08	0.66 (0.42–1.05)	107 (0.32)	0.15*
Allele C	180 (0.76)	98 (0.68)				229 (0.68)	

*All significant (*P*< 0.05–0.02) before correction for multiple comparisons (*P*_*c*_).

RXR-gamma: retinoid X receptor-gamma; OR: odds ratio; SNPs: single nucleotide polymorphisms; FCHL: familial combined hyperlipidemia.

The positions are related to NCBI Reference Sequence NC_000001.10 (Homo sapiens chromosome 1, GRCh37.p2 primary reference assembly).

**Table 3:** Haplotype frequencies in all FCHL affected and controls for the five analysed SNPs.

ID	Haplotypes	Case (*n* = 312)	Control (*n* = 156)	*P*_*c*_ value	Odds ratio [95% CI]
1	CGCGC	0.130 (41)	0.134 (21)	0.862126	1.052 [0.594~1.861]
2	CGTAG	0.070 (22)	0.077 (12)	0.913662	0.960 [0.463~1.994]
3	CGTGC	0.032 (10)	0.058 (9)	0.215309	0.558 [0.219~1.420]
4	CTCGC	0.077 (24)	0.09 (14)	0.780397	0.907 [0.456~1.804]
5	CTTAC	0.038 (12)	0.013 (2)	0.119377	3.081 [0.696~13.636]
6	CTTAG	0.189 (59)	0.115 (18)	**0.048031**	**2.020 [1.137~3.589]**
7	CTTGC	0.051 (16)	0.096 (15)	0.101978	0.547 [0.263~1.137]
8	TGTGC	0.054 (17)	0.135 (21)	**0.02361**	**0.386 [0.196~0.761]**
9	TTTGC	0.234 (73)	0.220 (34)	0.457251	1.194 [0.748~1.905]

CI: confidence interval; SNPs: single nucleotide polymorphisms; FCHL: familial combined hyperlipidemia.

All frequencies <0.03 have been ignored in analysis. Loci chosen for hap-analysis: rs1128977, rs2651860, rs2134095, rs283696, and rs10918169.

The number of chromosomes valid for analysis is reported in parentheses (312 out of 384 chromosomes for the 192 FCHL affected and 156 out of 238 chromosomes for the 119 controls). Global *χ* ^2^ is 18.905838 while df = 8. Fisher's *P* value is 0.015506; Pearson's *P* value is 0.015371.

## List of Abbreviations

RXRG: Retinoid X receptor gamma
FCHL: Familial combined hyperlipidemia
VLDL: Very-low-density lipoprotein
LDL: Low-density lipoprotein
LDL-C: Low-density lipoprotein-cholesterol
HDL-C: High-density lipoprotein-cholesterol
apoB: Apolipoprotein B
ALT: Alanine transaminase
AST: Aspartate transaminase
BMI: Body mass index
HOMA-IR: Homeostasis model assessment for insulin resistance
TG: Triglyceride
TC: Total cholesterol
PCR-RFLP: Polymerase chain reaction-restriction fragment length polymorphism
MAF: Minor allele frequency
LOD: Logarithm of the odds
IBD: Identical-by-descent
EDTA: Ethylenediaminetetraacetic acid
UTR: Untranslated region
UCSC: University of California, Santa Cruz
SNP: Single nucleotide polymorphism
LD: Linkage disequilibrium
OR: Odds ratio
CI: Confidence interval
SPSS: Statistical Product and Service Solutions
NS: Nonsignificant
HF: High fat
FXR: Farnesol X receptor
LXR: Liver X receptors
BAR: Bile acid receptor
PPAR: Peroxisome proliferator-activated receptor
*P*_*c*_: *P* value after applying Bonferroni's correction.
