# Supplementary material for: Association of RXR-Gamma Gene Variants with Familial Combined Hyperlipidemia: Genotype and Haplotype Analysis
Source: J Lipids. 2013 Oct 13;2013:517943. doi: 10.1155/2013/517943 (PMC3810489; doi:10.1155/2013/517943)
Supplement: Supplementary file 1 — Supplementary Table 1 shows the five genotyped SNPs of the RXR-gamma gene and their minor allele frequencies (MAF) in our controls. [file 517943.f1.pdf]

Supplementary Table 1. Genotyped SNPs of the RXR-gamma gene and their minor allele frequencies (MAF) in our controls in comparison with those reported in the NCBI database

Ref SNP ID	Location	Sequence Variation	Codon variation	MAF (our controls)
Rs1128977	Exon 3	C/T	Ala140Ala	0.38
Rs2651860	Intron 4	T/G	none	0.46
Rs2134095	Exon8	T/C	Val350Val	0.25
Rs283696	Intron 9	G/A	none	0.22
Rs10918169	Exon 10 3'UTR	G/C†	none	0.76

† Our data reported G-allele as the less frequent allele
